# Comparison on simultaneous caillary and venous parasite density and genotyping results from children and adults with uncomplicated malaria: a prospective observational study in Uganda

**DOI:** 10.1186/s12879-019-4174-1

**Published:** 2019-06-26

**Authors:** Aine Lehane, Moses Were, Martina Wade, Musleehat Hamadu, Megan Cahill, Sylvia Kiconco, Richard Kajubi, Francesca Aweeka, Norah Mwebaza, Fangyong Li, Sunil Parikh

**Affiliations:** 1 Yale School of Public Health, New Haven, CT USA; 2 Infectious Diseases Research Collaboration, Kampala, Uganda; 3 University of California-San Francisco, San Francisco, CA USA

**Keywords:** Malaria, Uganda, Capillary, Venous, Parasitemia, Genotyping, Complexity of infection, Microscopy, Strain diversity, Antimalarial, Parasite density

## Abstract

**Background:**

Blood smear microscopy remains the gold-standard method to diagnose and quantify malaria parasite density. In addition, parasite genotyping of select loci is the most utilized method for distinguishing recrudescent and new infections and to determine the number of strains per sample. In research settings, blood may be obtained from capillary or venous compartments, and results from these matrices have been used interchangeably. Our aim was to compare quantitative results for parasite density and strain complexity from both compartments.

**Methods:**

In a prospective observational study, children and adults presenting with uncomplicated *Plasmodium falciparum* malaria, simultaneous capillary and venous blood smears and dried blood spots were collected over 42-days following treatment with artemether-lumefantrine. Blood smears were read by two microscopists, any discrepancies resolved by a third reader. Parasite DNA fingerprinting was conducted using six microsatellites. Bland Altman analysis and paired t-test/McNemar’s test were used to assess the difference in density readings and measurements.

**Results:**

Two hundred twenty-three participants were included in the analysis (177 children (35 HIV-infected/142 HIV-uninfected), 21 HIV-uninfected pregnant women, and 25 HIV-uninfected non-pregnant adults). Parasite density measurements did not statistically differ between capillary and venous blood smears at the time of presentation, nor over the course of 42-day follow-up. Characterization of merozoite surface protein-2 (MSP-2) genetic polymorphism demonstrated a higher level of strain diversity at the time of presentation in venous samples, as compared with capillary specimens (*p* = 0.02). There was a high degree of variability in genotype-corrected outcomes when pairs of samples from each compartment were compared using MSP-2 alone, although the variability was reduced with the use of multiple markers.

**Conclusions:**

Parasite density measurements do not statistically differ between capillary and venous compartments in all studied demographic groups at the time of presentation with malaria, or over the course of follow-up. More strains were detected by MSP-2 genotyping in venous samples than in capillary samples at the time of malaria diagnosis. The use of multiple polymorphic markers reduces the impact of variability in strain detection on genotype-corrected outcomes. This study confirms that both capillary and venous compartments can be used for sampling with confidence in the clinical research setting.

**Trial registration:**

The trial was registered at ClinicalTrials.gov under registration no. NCT01717885.

**Electronic supplementary material:**

The online version of this article (10.1186/s12879-019-4174-1) contains supplementary material, which is available to authorized users.

## Background

The World Health Organization (WHO) estimated 219 million malaria infections and 435,000 deaths in 2017 with Africa accounting for 90% of these cases [1]. Uganda accounts for approximately 4% of total malaria cases worldwide with approximately 7.8 million cases in 2016 [1]. The gold standard for malaria diagnosis is examination of a Giemsa-stained blood film with light microscopy [2]. Blood films can be prepared using capillary or venous blood. In clinical settings, capillary finger-pricks are most commonly utilized for the detection and quantification of parasite density [2]. However, in research settings, either capillary or venous sampling may be employed over the course of the study, depending on the amount of blood needed at each visit. Notably, in these cases, venous and capillary smear results have been used interchangeably.

In treatment efficacy and other types of research studies, parasite DNA is typically extracted from blood samples, and genotyping of polymorphic markers using microsatellites is used to compare the parasite strains at the time of diagnosis to those present at the time of clinical or parasitological failure [3]. In addition, these same microsatellites are used to quantify the number of unique strains infecting an individual, which represents the complexity of infection (COI) [4]. Similar to parasite density measurements, parasite DNA fingerprinting results from either capillary or venous blood are used interchangeably.

Capillary and venous blood compartments have been studied for differences in measurements of hematocrit, leukocyte populations, glucose, G6PD-deficiency status, and other analytes [5–15]. Results of these studies vary, for example from finding slightly higher leukocyte counts in capillary than venous samples to platelet counts being higher in venous than capillary blood [5, 6, 8–10]. Notably, certain species of another intraerythrocytic parasite, *Babesia*, have been found to exhibit higher parasite density levels in capillary than in venous compartments [16, 17]. In the case of malaria, *Plasmodium falciparum* parasites are known to sequester in capillaries, a property which may impact the characterization of parasite density and/or strain dynamics in these matrices [18–21]. Indeed, a handful of studies, limited to asymptomatic or suspected malaria cases, have compared the two compartments [22–25]. While a majority have found no significant difference in the magnitude of asexual density in either compartment, a few studies have found a higher sensitivity for detection of asexual and gametocyte stages in capillary versus venous blood.

No data, to our knowledge, is available on the comparison of capillary and venous parasite density following treatment for symptomatic malaria, in pregnancy or in HIV-infected individuals, nor any data on comparing genotyping results from capillary and venous blood. We report the following findings with the goal of informing the interpretation of malaria parasite density and genotyping results from research studies.

## Methods

### Study area and patients

This study was part of a larger prospective pharmacokinetic/pharmacodynamics (PK/PD) study in Tororo, Uganda assessing PK/PD of artemether-lumefantrine for the treatment of uncomplicated malaria in the context of development, pregnancy, and HIV [26, 27]. Individuals from four groups (HIV- infected and HIV-uninfected children; HIV-uninfected non-pregnant adults; and HIV-uninfected pregnant women) were enrolled if presenting with uncomplicated malaria (defined as fever [tympanic temperature of ≥38 °C] or history of fever within the past 24 h) and *P. falciparum* parasitemia as detected by capillary thick blood smear [26, 27]. Exclusion criteria included severe malaria, as defined by the WHO [28], presence of significant other illnesses such as tuberculosis, or antimalarial drug treatment within 2 weeks prior to study enrollment [26]. Enrollment was from August 2011 to November 2014 for children and February 2013 to December 2014 for adults.

### Sample collection

**Fig. 1:**
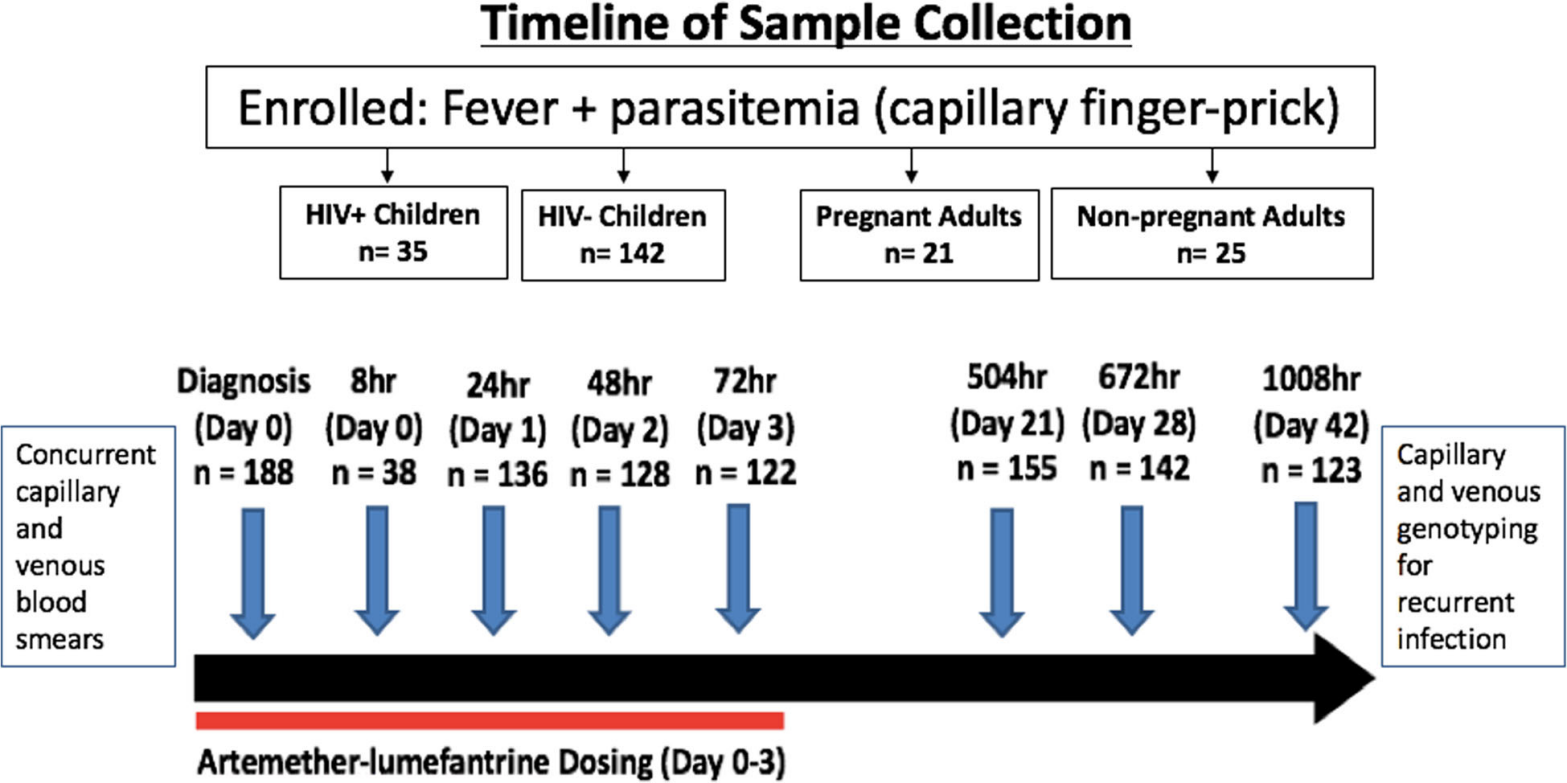
Timeline scheme for blood collection. Simultaneous capillary and venous samples were collected at multiple time points over the course of 42-day follow-up

In the context of a prospective observational study of children and adults presenting with uncomplicated *P. falciparum* malaria, simultaneous capillary and venous blood smears and dried blood spots were collected at several time points over 42-day follow-up following treatment with artemether-lumefantrine which comprised of six doses administered as per weight-based guidelines beginning on day 0 with most doses administered twice daily. Enrolled study participants were clinically evaluated in the study clinic with active and passive surveillance on study days 0, 1, 2, 3, 4, 8, 14, 21, 28, and 42 (Fig. 1). Capillary blood was obtained by finger-prick and placed directly onto filter paper. Venous blood was obtained by antecubital venipuncture into EDTA, and immediately placed onto filter paper by pipetting. Blood volume was added to fill pre-specified diameter blood spots.

### Parasite densities

Giemsa-stained thin and thick blood smears were evaluated for speciation and to quantify parasite density, respectively. Parasite densities were calculated by counting the number of asexual parasites per 200 leukocytes, assuming a leukocyte count of 8000/μL. A blood smear was declared negative when no asexual parasites were seen under 100 high-power fields [26]. All blood films were air-dried and stained with 2% Giemsa. The blood films were read by two expert microscopists. In the case of any discrepancies with the results a third microscopist was brought in and that result was considered final.

### Genotyping

Dried blood spots were collected on filter paper (Whatman 3MM; Whatman, Clinton, NJ), stored at 4 °C and DNA was extracted from a single DBS spot, using the entire pre-specified circle, using the QIAamp DNA Mini Kit (Qiagen, Valencia, CA). DNA was used to distinguish new infections from recrudescent ones for all recurrent episodes of malaria, using previously described methods [29]. Genotyping was done in a stepwise fashion using six polymorphic markers, two of which were merozoite surface proteins (*msp1* and *msp2*) and four microsatellite markers (TA40, TA60, TA81, and TA87) [29]. Genotyping for each sample was completed by doing PCR-based amplification, followed by capillary electrophoresis [29]. If, for any of the markers, an allele was not shared between consecutive episodes of parasitemia, then the episode was considered as a new infection; if one allele or more was shared at all six loci, then the episode was classified as recrudescent [26].

### Statistical analysis

Descriptive statistics such as frequency, median and range were presented to summarize study population. Paired t-test or McNemar’s test were used to assess the statistical significance of the difference between two measurements. To assess the agreement of density readings between the two methods, Bland Altman analysis was conducted. Logarithmic transformation was carried out due to the skewed distribution of parasite density values. Then Bland Altman plots were generated as the average against the difference of the two measurements. The 95% limit of agreement was reported as a measure of agreement. Cohen’s kappa coefficient was used to test agreement of positive and negative parasitemia readings, and Spearman’s correlation of density and number of strains was also performed. Statistical significance was set at *p* < 0.05. The data were analyzed using Stata version 12.1 (StataCorp, College Station, TX, USA) and SAS version 9 (SAS Institute, Cary, NC, USA). Statistical analysis was performed in conjunction with Yale Center for Analytical Sciences.

## Results

### Demographic characteristics of study population

A total of 223 participants were included in the analysis. This included 177 children (35 HIV-infected children and 142 HIV-uninfected children), 21 HIV-uninfected pregnant women, and 25 HIV-uninfected non-pregnant adults. The male to female ratio for children was 1:1 and the median age at time of presentation overall was 3.8 years (range 1.2–8.5); for HIV-infected, 5.6 years (range 2.2–8.5), and for HIV-uninfected, 3.5 years (range 1.1–7.9). The male to female ratio for non-pregnant adults was 1:2 and the median age was 24.9 years (range 16.1–67.9). The median age for pregnant women was 24.4 years (range 17.8–34.9). 214 out of 223 participants had 42-day treatment outcomes, with 108/213 (50.5%) having recurrent parasitemia by day 42 of follow-up.

### Parasite density measurements

**Fig. 2:**
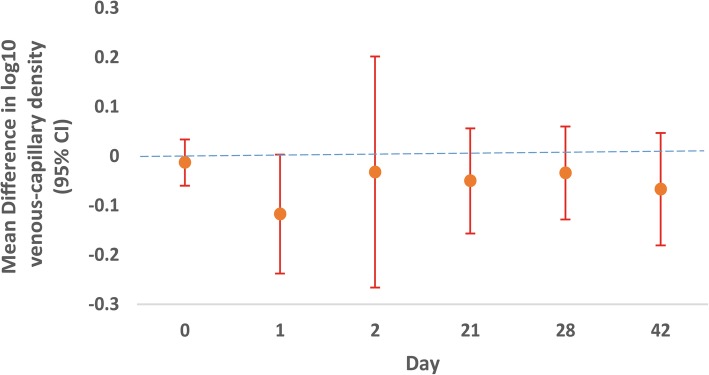
Mean difference in capillary and venous parasite density at multiple time points during follow-up. The mean difference in parasite density measurements do no statistically differ between capillary and venous over the course of 42-day follow-up except for borderline significance with capillary higher than venous by 21% on day 1 (p = 0.056). Parasite densities represented on a log10 scale

Parasite density (parasites/μL) measurements from simultaneous capillary and venous blood smears were not statistically different at the time individuals presented with uncomplicated *P. falciparum* malaria, nor over the course of follow-up except for borderline significance with capillary higher than venous specimens by 21% on day 1 (*p* = 0.056), when aggregating all demographic groups (Fig. 2; Additional file 1: Table S1). This lack of difference was true for children, adults, and pregnant women separately as well. Table 1 shows the breakdown of overall parasite density results by demographic group for day 0 and day 1. When comparing day 0 parasite densities between children, non-pregnant adults, and pregnant women, venous densities significantly differed by these three groups (*p* < 0.05), with children having the highest densities and non-pregnant adults having the lowest densities. Likewise, capillary readings were significantly greater in children compared to readings in pregnant women or non-pregnant adults *(p < .*001).

**Table 1:** Parasite density comparison for day 0 and day 1 in children, non-pregnant adults, and pregnant women

Days	N	Capillary smear, Geometric Mean (Geometric SD)	Range: min-max	N	Venous smear, Geometric Mean (Geometric SD)	Range: min-max	Relative Difference (ratio)	*p*-value
Children								
0	152	18,503 (7.4)	(32.0, 378E3)	152	18,996 (7.6)	(16.0, 368E3)	1.0 (0.9, 1.2)	0.65
1	81	615.6 (10.6)	(16.0, 60,800)	76	548.3 (11.9)	(16.0, 41,600)	0.80 (0.6, 1.1)	0.14
Non-pregnant adults								
0	21	497.9 (10.4)	(32.0, 22,080)	21	410.9 (12.8)	(16.0, 44,800)	0.8 (0.5, 1.3)	0.38
1	5	242.5 (6.4)	(16.0, 1280)	4	414.4 (2.6)	(192.0, 1600)	0.9 (0.2, 3.6)	0.77
Pregnant women								
0	15	6800 (5.0)	(480.0, 60,160)	15	4703 (5.8)	(64.0, 51,200)	0.7 (0.5, 1.0)	0.06
1	5	215.1 (3.9)	(48.0, 640.0)	5	68.5 (4.2)	(16.0, 480.0)	0.3 (0.1, 1.8)	0.14

Geometric mean parasite density measurements, range, and relative difference results for children, non-pregnant adults, and pregnant women on day 0 and day 1 for both capillary and venous compartments

**Fig. 3:**
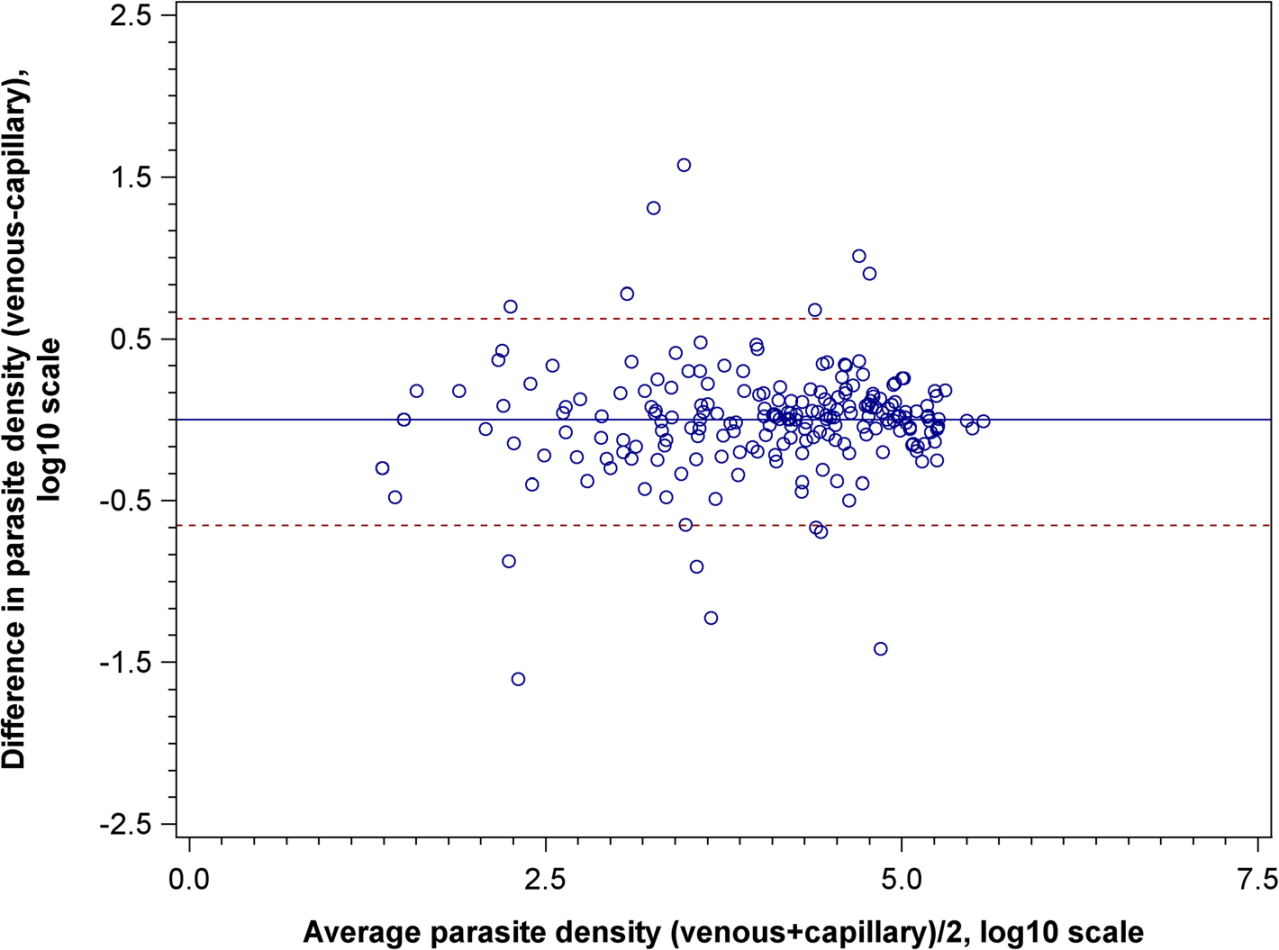
Bland Altman plot of capillary and venous day 0 parasite density measurement agreement. The difference in simultaneous capillary and venous parasite density measurements on day 0 can be as large as 1.5 log_10_-fold based on the 95% limit of agreement. The difference may be in either direction, and the variability does not change with magnitude of measurements. Parasite densities represented on a log10 scale

McNemar’s test was used to compare the two compartments for zero values for parasite density measured following the day of diagnosis, revealing no significant difference, aside from a borderline difference on day 1 (30.5% zero values in capillary vs. 35.1% in venous, *p* = 0.06). This result is consistent with results for non-zero parasite density measurements in Fig. 2 and Additional file 1: Table S1. Additionally, the Bland Altman plot (Fig. 3) shows that the difference in simultaneous capillary and venous parasite density measurements on day 0 can be as large as 1.5 log_10_-fold based on the 95% limit of agreement. However, the difference may be in either direction, and the variability does not change with magnitude of measurements.

On the day that recurrent parasitemia (with or without fever) was detected, simultaneous capillary and venous blood smears were available for *n* = 70 individuals. Geometric mean parasite densities were 2072 (95% CI 1116, 3850) and 2499 parasites/uL (95% CI 1412, 4422) in capillary and venous blood smears at the time of recurrent parasitemia, respectively, which was not statistically different (*p* = 0.96).

### Complexity of infection based on MSP-2 and other polymorphic markers

**Fig. 4:**
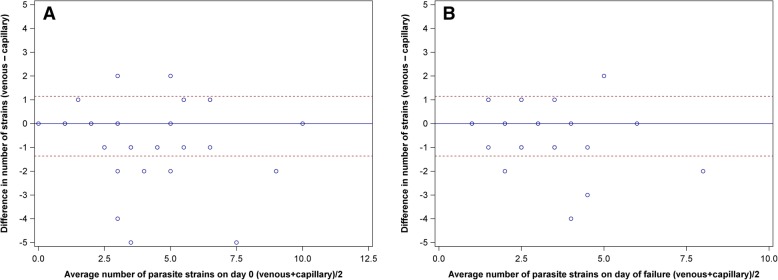
Bland Altman plots of agreement between venous and capillary number of strains based on MSP-2 genotyping. Bland Altman plots of agreement for number of strains based on marker MSP-2 on Day 0 (A) and day of failure (B). More strains were detected in venous samples than capillary on day 0 (p = 0.02) but not at day of failure, using the MSP-2 marker

Based on genotyping of MSP-2 alone, the most common marker for COI determination, venous samples had a statistically higher number of distinct strains detected than capillary samples on the day of diagnosis (*p* = 0.02), but not at the time of recurrent malaria (*p* = 0.12). Bland Altman plots (Fig. 4) show the 95% limits of agreement between number of strains for the MSP-2 marker alone on day of diagnosis and day of failure. A significant difference in number of strains was not detected in the remaining five markers. Additionally, in both compartments, the number of strains detected did not correlate with parasite density on day 0 (Spearman correlation r = 0.10 and 0.03; *p* = 0.53 and 0.82 for capillary and venous compartments, respectively).

### Genotyping to distinguish new infections from recurrent infections

Out of the 223 participants, 214 had 42-day outcomes available, with recurrent malaria (either parasitemia or parasitemia plus fever) occurring in 50.5% (108/214) over the course of 42-day follow-up. Of these cases, simultaneous capillary and venous genotyping samples were available in *n* = 52 participants at both day 0 and the day of failure. In these cases, MSP-2-based genotyping to distinguish recrudescent from new infections showed a high degree of variability depending on the pairing of compartments on the day of presentation and the day of recurrent malaria (Tables 2 and 3). When using the capillary sample for both day 0 and the day of failure, 19% of infections would have been classified as recrudescent, as compared to 30%, when a day 0 capillary sample was paired to a venous sample on the day of failure. The agreements between genotyping calls for MSP-2 from different possible pairing of compartments vary as indicated using Cohen’s Kappa coefficient (Table 3). The agreement between the use of capillary/capillary samples and the use of venous/venous samples is moderate, with the least Kappa of 0.45, while the agreement between the use of capillary/venous and the use of venous/capillary is excellent (Kappa 0.84). However, the use of multiple markers (MSP-1, MSP-2, and four microsatellites) greatly reduced both the reported proportion of recrudescent infections (Table 2). Each compartment frequently detects strains that are not seen in other compartment. Out of 104 samples with paired simultaneous capillary and venous genotyping results, 54.9% (57/104) showed discrepancies in the strains detected between matrices using MSP-2 capillary electrophoresis.

**Table 2:** Genotyping calls of infection as recrudescent or new infection based on one or more markers. PCR-corrected treatment outcomes based on MSP-2 or all six molecular markers

	Marker	Capillary-Capillary	Capillary-Venous	Venous-Venous	Venous-Capillary
% Recrudescence (n)	MSP- 2 alone	19% (9)	30% (14)	25%(12)	27% (13)
	All 6 markers	5.8% (3)	4% (2)	2% (1)	4% (2)

Genotyping calls based on MSP-2 alone or all six markers, in the four possible compartment combinations on day 0 and day of failure. There is a high degree of variability in genotyping calls between pairs of samples from each compartment using MSP-2 alone, although the variability is reduced with the use of multiple markers

**Table 3:** Agreement between MSP2 genotype calls between various combinations of capillary and venous samples

	Capillary-Venous	Venous-Venous	Venous-Capillary
Capillary-Capillary	0.71 (0.49–0.94)	0.45 (0.15, 0.75)	0.77 (0.55, 0.98)
Capillary-Venous	–	0.68 (0.45, 0.92)	0.84 (0.67, 1.00)
VV	–	–	0.62 (0.36, 0.87)

Simple Kappa coefficient and 95% confidence interval. Capillary-Capillary (capillary day 0 compared to capillary day of failure); Capillary-Venous (capillary day 0 compared to venous day of failure); Venous-Venous (venous day 0 compared to venous day of failure); Venous-Capillary (venous day 0 compared to capillary day of failure)

## Discussion

Our study showed that microscopy-based parasite density measurements from simultaneous capillary and venous blood are not systematically different from one another for patients presenting with uncomplicated *P. falciparum* malaria. While there is a large variability between measurements in the two compartments, the variability may be in either direction and is evident over a wide range of parasite density values. In the case of genotyping, the use of a single marker (MSP-2) demonstrated a higher level of strain diversity in venous blood compared to capillary blood on the day of diagnosis and would have demonstrated between 19 to 30% rate of recrudescence depending on the pairing of sample compartments.

Our initial hypothesis was that systematic differences in parasite density may be present between matrices given that *Plasmodium falciparum* parasites are known to sequester in capillaries [18–21]. In addition, we hypothesized that pregnant women may demonstrate differences from other demographic groups, due to changes in hemodynamic parameters during pregnancy [30]. However, measurements in clinical malaria from capillary and venous compartments did not demonstrate a significant difference from one another throughout the study time points, aside from a slightly higher parasite density in capillary versus venous specimens by 21% on Day 1 (*p* = 0.056), a magnitude which is not of clinical significance. This lack of difference was seen in all demographic groups.

Our results are largely in alignment with previous studies, however, there are some notable differences (see summary of studies in Additional file 1: Table S2) [23–25, 31]. Similar to our results, a lack of difference in asexual parasite density measurements was reported in two distinct Cameroonian studies, one including 137 asymptomatic gametocyte-positive Cameroonian children (age 4–15 years), and another involving 150 Cameroonians presenting with symptoms of malaria (ages 7 to 66 years) [22, 23]. However, two studies have reported differences, although the magnitude of differences was small. To our knowledge, the first study to compare parasite density in both compartments was conducted in Burkina Faso in 73 asymptomatic children, where densities were found to be statistically higher in capillary than in venous blood compartments (median parasitemia capillary and venous, 300 and 200 parasites/uL, respectively [24]. The most recent study to explore this was in Gabon, and similarly found a higher median asexual density in those presenting with symptoms of malaria [25]. However, parasite densities were low in this study, with minor differences between compartments (median parasitemia capillary and venous, 495 and 429 parasites/uL, respectively) that are of unclear clinical significance. One fairly consistent finding, however, is that the detection of parasites may be more sensitive in capillary versus venous blood in those presenting with low initial parasite density [22, 24, 25]. In the Cameroonian study, those presenting with symptoms of malaria had positive capillary smears in 29% of cases, but only 17% of venous smears (*p* = 0.011), while the Gabon study found that capillary blood had an 8% higher sensitivity than venous blood [22, 25]. While our study was not powered to detect this outcome, at the time of recurrent parasitemia, 4 samples were positive only in the capillary compartment, and one sample only in the venous compartment. Nonetheless, overall parasite density did not differ between compartments at the time of detection with recurrent parasitemia.

Another aspect which has been compared in these compartments is the ability to detect gametocytes. *P. falciparum* gametocyte densities, as determined by microscopy did not differ between capillary and venous blood in the Cameroonian study of asymptomatic children [23], but an exploratory study in Ethiopia found a trend towards a higher absolute count of Pfs25-mRNA copies in capillary blood, and the Gabon study found both a higher sensitivity for gametocyte detection and higher densities in capillary versus venous blood by microscopy, both involving symptomatic individuals [31]. Thus, additional studies are needed, but data may suggest that capillary specimens have higher gametocyte loads than venous samples at the time of presentation with symptomatic malaria, when overall parasite densities are higher. Such data may have implications on our understanding of malaria transmission.

Thus, in totality, results support either a lack of difference or slightly higher asexual parasite density in capillary as compared to venous blood. Furthermore, while there may be large differences in the magnitude of density between the compartments, these differences are not consistently in one direction, and do not appear to be of a magnitude that is clinically relevant. Additionally, the magnitude in variability in parasite density measurements may be in line with other sources of variability such as inter-observer agreement among microscopists [32–34]. Indeed, previous studies of intra-observer agreement from microscopy have revealed up to a 17% median discrepancy in parasite counts when two microscopists examined the same slide [34]. Thus, in comparing the magnitude of variability in our results, with those seen in studies of readings between microscopists, we do not think the variability in parasite density measurements between the two compartments is of clinical significance. Additional areas warranting further exploration are the possibility of different sensitivities for detection of low density asexual parasite and gametocyte densities in capillary compared to venous blood samples.

To our knowledge, we also present the first data on the comparison of genotyping results from capillary and venous blood [3]. The use of MSP-2-based genotyping alone, we found venous samples had a statistically higher number of strains detected than capillary samples on the day of diagnosis (*p* = 0.02), but not at the time of recurrent malaria (*p* = 0.12), when parasite densities were comparatively lower than at initial presentation. The explanation for the higher number of strains detected in venous than in capillary blood by MSP-2, the marker demonstrating the highest level of polymorphism in our study (data not shown) is unclear and will require further study to see if results are reproducible in other settings.

Finally, we sought to assess whether differences in genotyping results from capillary and venous blood impact PCR-corrected outcome classifications. While the WHO recommends the use of multiple markers (typically MSP-1, MSP-2, and GLURP), a large number of studies continue to use MSP-2-based genotyping alone [3]. In our study, genotype-corrected outcomes displayed a high degree of variability depending on the pairing of compartments on the day of presentation and the day of failure, ranging from 19 to 30%. We currently use a step-wise approach of six markers (MSP-2, MSP-2, and 4 microsatellites) to assess for recrudescent malaria, and, not surprisingly, the use of these additional markers greatly reduced both the reported proportion of recrudescent infections and the variability in calls depending on the pairing of sample compartments (2–5.9%).

Our study has a few limitations. Notably, we identified individuals for enrollment based on the presence of a fever with a capillary blood smear that was positive for parasites. Thus, we would have missed individuals who were positive only by venous smear at diagnosis. In addition, we did not include any asymptomatic or sub-patent infections. Additionally, our study took place in a region with high transmission intensity and strain diversity. It is likely that strains may have been missed due to known limitations of PCR-based genotyping in multi-clonal infections. Lastly, the use of PCR-based genotyping from dried blood spots has inherent limitations in the sensitivity, due both to the technique, as well as the limitation of blood volume. It is likely that a larger blood volume and/or newer sequence-based techniques may reveal other patterns not seen at our level of discrimination.

## Conclusions

Quantitative parasite density measurements do not statistically differ between capillary and venous compartments in children, adults, or pregnant women at the time of presentation with uncomplicated malaria, nor over the course of standard 42-day follow-up. However, more strains were detected by MSP-2 genotyping in venous samples than in capillary samples at the time of diagnosis. The variability in genotype-corrected treatment outcomes is high when using a single marker, but is dramatically reduced by the use of multiple polymorphic markers. These findings indicate that both capillary and venous compartments can be used for sampling with confidence in clinical research settings.

## Additional file

Additional file 1: **Table S1.** Mean difference in parasite density (parasites per μL) at different time points over the course of follow-up in all participants. **Table S2.** Summary of studies comparing capillary and venous measurements for malaria parameters. (DOCX 26 kb)

## Data Availability

The data are available from the corresponding author upon reasonable request.

## Abbreviations

COI: Complexity of infection
DNA: Deoxyribonucleic acid
G6PD: Glucose-6-phosphate dehydrogenase
HIV: Human immunodeficiency virus
MSP: Merozoite surface protein
PCR: Polymerase chain reaction
PK/PD: Pharmacokinetic/pharmacodynamics
WHO: World Health Organization
