# Supplementary material for: Comparison on simultaneous caillary and venous parasite density and genotyping results from children and adults with uncomplicated malaria: a prospective observational study in Uganda
Source: BMC Infect Dis. 2019 Jun 26;19:559. doi: 10.1186/s12879-019-4174-1 (PMC6595677; doi:10.1186/s12879-019-4174-1)
Supplement: Supplementary file 1 — Table S1. Mean difference in parasite density (parasites per μL) at different time points over the course of follow-up in all participants. Table S2. Summary of studies comparing capillary and venous measurements for malaria parameters. (DOCX 26 kb) [file 12879_2019_4174_MOESM1_ESM.docx]

**Supplemental Table 1: Mean difference in parasite density (parasites per µL) at different time points over the course of follow-up in all participants**

|  |  | Capillary smear | | Venous smear | | p-value | Mean ratio  venous/capillary  (95% limit of agreement) |
| --- | --- | --- | --- | --- | --- | --- | --- |
| Days | N | Geometric Mean (Geometric SD) | Range | N  Geometric Mean (Geometric SD) | Range |  |  |
| 0 | 188 | 11407 (10.0) | 32-377,000 | 11070 (11.0) | 16-369,535 | 0.58 | 1.00 (0.2, 4.2) |
| 8 hrs | 38 | 4024 (8.2) | 32- 88,433 | 4447 (7.4) | 16-86,682 | 0.66 | 1.00 (0.7, 1.4) |
| 1 | 91 | 552 (9.9) | 16-6,1084 | 478 (11.2) | 16-41,773 | 0.05 | 0.8 (0.1, 9.2) |
| 2 | 14 | 75 (4.9) | 16-1,120 | 72.8 (3.7) | 16-520 | 0.76 | 0.9 (0.2, 4.5) |
| 21 | 22 | 1434 (11.4) | 16-59,874 | 1328 (8.6) | 32-82,454 | 0.33 | 0.9 (0.3, 2.5) |
| 28 | 33 | 2972 (9.8) | 16-65,513 | 3124 (9.9) | 16-74,608 | 0.46 | 0.9 (0.3, 3.0) |
| 42 | 22 | 1477 (11.4) | 16-68,872 | 1316 (10.5) | 16-66,171 | 0.23 | 0.09 (0.3, 2.6) |

Legend: Geometric mean difference in parasite density between capillary and venous compartments in cases with parasitemia above zero

**Supplementary Table 2. Summary of studies comparing capillary and venous measurements for malaria parameters**

| **Location** | **Age Group** | **n** | **Clinical Status** | **Time point** | **Method** | **Capillary vs Venous Comparison** | **Ref** |
| --- | --- | --- | --- | --- | --- | --- | --- |
| Uganda | All ages, pregnancy | 223 | Symptomatic malaria | Multiple | Microscopy, PCR genotyping | No difference in asexual density, higher MSP-2 diversity in venous than capillary | Current study |
| Burkina Faso | Adults | 73 | Asymptomatic parasitemia | Single | Microscopy | Capillary more sensitive and higher density than venous sample | [1] |
| Cameroon | All ages | 150 | Symptoms of malaria | Single | Microscopy | No difference in asexual density, but higher sensitivity of capillary vs venous detection of asexual parasitemia | [2] |
| Cameroon | Children | 137 | Asymptomatic, gametocyte positive | Single | Microscopy | No difference asexual or gametocyte density by microscopy | [3] |
| Ethiopia | Adults | 15 | Symptomatic malaria | Single | mRNA | Possible higher capillary Pfs25 copy number in capillary vs venous blood | [4] |
| Gabon | All ages | 346 | Symptoms of malaria | Single | Microscopy (qPCR gold-standard) | Slightly higher median asexual density, and higher sensitivity for gametocyte and asexual detection in capillary vs venous blood | [5] |

References

1. Ouédraogo J, Lamizana L, Toe A, Kumilien S, Gbary A, Guigemdé T: **Emergence du paludisme chimiorésistant au Burkina Faso**. *Médecine d’Afrique Noire* 1991, **38**(4):275-278.

2. Njunda A, Assob N, Nsagha S, Kamga F, Mokenyu M, Kwenti E: **Comparison of capillary and venous blood using blood film microscopy in the detection of malaria parasites: A hospital based study**. *Scientific Journal of Microbiology* 2013, **2**(5):89-94.

3. Sandeu MM, Bayibéki AN, Tchioffo MT, Abate L, Gimonneau G, Awono-Ambéné PH, Nsango SE, Diallo D, Berry A, Texier G *et al*: **Do the venous blood samples replicate malaria parasite densities found in capillary blood? A field study performed in naturally-infected asymptomatic children in Cameroon**. *Malaria Journal* 2017, **16**(1):345.

4. Kast K, Berens-Riha N, Zeynudin A, Abduselam N, Eshetu T, Löscher T, Wieser A, Shock J, Pritsch M: **Evaluation of *Plasmodium falciparum* gametocyte detection in different patient material**. *Malaria journal* 2013, **12**(1):438.

5. Mischlinger J, Pitzinger P, Veletzky L, Groger M, Zoleko-Manego R, Adegnika AA, Agnandji ST, Lell B, Kremsner PG, Tannich E *et al*: **Use of Capillary Blood Samples Leads to Higher Parasitemia Estimates and Higher Diagnostic Sensitivity of Microscopic and Molecular Diagnostics of Malaria Than Venous Blood Samples**. *J Infect Dis* 2018, **218**(8):1296-1305.
